# Management of Local Stressors Can Improve the Resilience of Marine Canopy Algae to Global Stressors

**DOI:** 10.1371/journal.pone.0120837

**Published:** 2015-03-25

**Authors:** Elisabeth M. A. Strain, Jim van Belzen, Jeroen van Dalen, Tjeerd J. Bouma, Laura Airoldi

**Affiliations:** 1 University of Bologna, Dipartimento di Scienze Biologiche, Geologiche ed Ambientali, Ravenna, Italy; 2 Royal Netherlands Institute for Sea Research, Spatial Ecology Department, Yerseke, Netherlands; Università di Genova, ITALY

## Abstract

Coastal systems are increasingly threatened by multiple local anthropogenic and global climatic stressors. With the difficulties in remediating global stressors, management requires alternative approaches that focus on local scales. We used manipulative experiments to test whether reducing local stressors (sediment load and nutrient concentrations) can improve the resilience of foundation species (canopy algae along temperate rocky coastlines) to future projected global climate stressors (high wave exposure, increasing sea surface temperature), which are less amenable to management actions. We focused on Fucoids (*Cystoseira barbata*) along the north-western Adriatic coast in the Mediterranean Sea because of their ecological relevance, sensitivity to a variety of human impacts, and declared conservation priority. At current levels of sediment and nutrients, *C*. *barbata* showed negative responses to the simulated future scenarios of high wave exposure and increased sea surface temperature. However, reducing the sediment load increased the survival of *C*. *barbata* recruits by 90.24% at high wave exposure while reducing nutrient concentrations resulted in a 20.14% increase in the survival and enhanced the growth of recruited juveniles at high temperature. We conclude that improving water quality by reducing nutrient concentrations, and particularly the sediment load, would significantly increase the resilience of *C*. *barbata* populations to projected increases in climate stressors. Developing and applying appropriate targets for specific local anthropogenic stressors could be an effective management action to halt the severe and ongoing loss of key marine habitats.

## Introduction

Many valuable marine ecosystems are in decline due to the combined effects of multiple local anthropogenic and global climatic stressors [1, 2]. The interactions between these stressors can cause declines in foundation species and loss of critical habitats [3, 4, 5]. Ensuring the resilience of these habitats requires developing management strategies to mitigate the effects of multiple stressors and increase the resilience (i.e. the capacity to recover from a disturbance) of foundation species [6, 7, 8]. Global stressors, such as rising sea surface temperature, ocean acidification and increasing storminess, operate at large spatial scales, are driven by complex processes, and developing management strategies for these stressors requires the collaboration of multiple countries [6]. Therefore, management actions aimed at mitigating multiple stressors tend to focus on reducing local stressors such as, improving water quality and limiting erosion or beach nourishments [6, 9].

The interactions between local and global stressors can complicate the development of management strategies [6]. Stressors can have additive impacts (the response is equal to the sum of individual stressors) and non-additive synergistic and antagonistic effects (the response is greater or less than the sum of individual stressors, respectively) on the growth and/or survival of foundation species [4, 10]. Although knowledge of the interactions between local and global stressors is increasing [3, 4, 5, 11], relatively little attention has been given to understanding the role of managing local stressors in disrupting these interactions [6, 12]. Current thinking suggests that when the interactions are additive or synergistic, reducing the local stressor(s) could be of benefit [4]. Implicit in these ideas is that effective management of one or more local stressors is crucial for improving the resilience of foundation species to global stressors [5]. However, modelling studies have questioned this assumption [8], raising the need for experimental tests of this hypothesis [12].

Canopy algae are foundation species that control structural complexity, productivity, nutrient cycling and biodiversity in temperate rocky reefs [13, 14]. Globally, loss of canopy forests has been reported in several regions, particularly in urban areas [1, 15, 16, 17]. These lost canopies are often replaced by species of lesser structural and ecological value including turf-forming, ephemeral or encrusting algae [13, 18, 19]. Such habitat shifts have been attributed to the complex interactions between local and global stressors [5, 11]. Recent meta-analyses for canopy algae, have shown that the majority of the interactions between these stressors were either additive or synergistic [5, 11]. This has motivated further research aimed at identifying effective local management options to prevent these habitat shifts [7, 20].

Our objective was to assess whether management actions aimed at reducing local anthropogenic stressor(s) could increase the resilience of foundation species to global climatic stressors, using canopy algae as a model system. We focused on *Cystoseira barbata* C. Agardh (Fucales: Sargassaceae) from the highly urbanised north-western Adriatic coastline in the Mediterranean Sea. Populations of fucoid algae are declining in this region [21] and elsewhere in the Mediterranean Sea [15, 22, 23, 24] due to a combination of multiple stressors. We focused on two local stressors of particular concern in the region sedimentation from beach erosion, dredging and nourishments [25]; and nutrient enrichment from sewage and agricultural runoff [26, 27, 28]. In this area there are relatively few grazers or other biological stressors [21]. Worldwide, these two stressors have been identified as key drivers in the loss of canopy algae, particularly in enclosed seas or catchments, such as the Baltic Sea [29, 30], North Sea [31], several bays around Australia [32, 33] and the Mediterranean Sea [34, 35]. Previous studies have suggested there is an additive interaction between high sediment load and wave exposure [36, 37] and a synergistic interaction between declining water quality (high sediment load and nutrient concentrations) and rising sea surface temperature [33]. However, experiments testing the effects of reducing these stressors are lacking. We used manipulative experiments to test whether reducing the sediment load would lead to increased growth and survival of the early life stages of *C*. *barbata* at high wave exposure, and if reducing both the sediment load and nutrient concentrations would enhance its growth and survival with rising sea surface temperature.

## Materials and Methods

### Ethics statement

Permission to work at La Vela, Monte Conero was granted by the Numana Guardia Costiera. This study did not involve any endangered or protected species. Unlike many other species in the genus, *Cystoseira barbata* is not classified as a protected species [38].

### Study site

We conducted 3 manipulative experiments using recruits and juveniles of *C*. *barbata* from La Vela located on the Monte Conero promontory (43°33N, 13°37E), in the north-western Adriatic Sea (Table 1). This site hosts the only significant *C*. *barbata* population in the Monte Conero promontory and is one of the few in the north-western Adriatic Sea [21]. *C*. *barbata* at La Vela has a strongly seasonal recruitment (late April to late May) and growth season (early June to early October) and can be found at depths ranging between 2 and 5 m [21].

**Table 1:** Overview of the experimental treatments and levels.

Treatment levels	Ambient	Reduced	Ambient	High
**Experiment 1**				
Sediment load	3000 g m^−2^	Weekly clearings (20 ± 5% reduction)		
Wave exposure			0.027–0.183 m relative height	0.04–0.261 m relative height
**Experiment 2**				
Sediment load	3000 g m^−2^	750 g m^−2^		
Wave exposure			0 m relative height	0.16–0.33 m relative height
**Experiment 3**				
Nutrient concentrations	DIN = 15 μmol l^−1^, PO_4_ = 0.15 μmol l^−1^	DIN < 5 μmol l^−1^, PO_4_ < 0.05 μmol l^−1^		
Sediment load	3000 g m^−2^	750 g m^−2^		
Temperature			24°C	27°C

The rocky reefs at La Vela are comprised of large limestone boulders. For Experiment 1, we attached marble tiles onto some of these boulders to collect recruits while in Experiment 2 and 3 juveniles were collected directly from open boulders (see below for details) which were located nearby to the canopy forest. The early life stages of *C*. *barbata* have a low survival probability at La Vela [21], therefore our collections were unlikely to lead to major declines in the abundance of the source population.

### Measurements of sediment and nutrients

Between late April and early October 2012, we monitored sediment deposition and accumulation, and the concentration of nutrients in seawater in the canopy forests at La Vela (S1 Fig.). All samples were collected between 2.5 and 3 m depth. To measure sediment deposition at the site we attached sediment traps 51 mm diameter, 200 mm high, aspect ratio 3.9 (n = 2) to flat rocks using epoxy putty (Subcoat S, Veneziani). Traps were deployed for periods of 18–53 days, depending on weather conditions. Traps were closed with plastic stoppers before retrieval. The accumulated sediment was collected in 0.1 × 0.1 m quadrats, in a standard 10 minute period in April and September using a suction sampler (n = 8). The sediment collected in each trap and quadrat was filtered through a 63 μm mesh sieve, rinsed with distilled water to remove salts and dried at 80°C for 7 days. The sediments were sorted into fine (63–250 μm) and coarse (250–1000 μm) size classes before weighing. Concentrations of nutrients (NO_3_, NO_2_, NH_4_, PO_4_) were measured in samples of seawater collected in polyethylene bottles (50 ml), monthly (n = 2). These samples were immediately filtered through Whatman GF/C filters and returned to the laboratory. All samples were processed using a Hach DR 2010 Spectrophotometer within 12 hours of collection. The sediment used in Experiments 2 and 3 was collected in April 2012 using a suction sampler and processed using the same technique as described above. The amount of sediment and nutrients applied to the ambient treatments in Experiments 2 and 3 were based on studies conducted in the Monte Conero region between 2008 and 2011 [28, 34], (S1 Table, S2 Fig.). We compared our measurements of the accumulated sediment and nutrient concentrations at La Vela in 2012 to the ambient treatments applied in Experiments 2 and 3 using t-tests. We also compared the measurements at La Vela taken in 2012 to other data collected in the Monte Conero region between 2008 and 2011 [28, 34], (S1 Table, S2 Fig.).

### Experiment 1: Effects of reducing the sediment load at different levels of wave exposure on recruits *in situ*

We ran a 3 month field experiment to test the benefits of reducing the sediment load at different levels of wave exposure on *C*. *barbata* recruits. In late April 2013, we attached 16 marble tiles (100 × 100 × 10 mm), ~500 mm apart, in 2.5–3 m depth, 8 tiles in front of (high wave exposure treatment) and 8 tiles behind (low wave exposure treatment) large boulders using epoxy putty (Subcoat S, Veneziani). Each of the 8 tiles (in the high and low wave exposure positions) was randomly assigned to either ambient or reduced sediment treatments (resulting in n = 4 replicate tiles for each wave exposure × sediment treatment).

Differences in wave exposure between the high and low wave exposure treatments were quantified by attaching gypsum balls (n = 3) onto rocks at both positions for 24 hours, in May and June. All gypsum balls were dried for 48 hours at 80°C and weighed, before and after their deployment. There was 71% higher mass loss in the high wave exposure treatment (mean = 2.318 g, SE = ± 0.27) relative to the low wave exposure treatment (mean = 0.921 g, SE = ± 0.07). The relative wave height (significant wave height/depth) in the low wave exposure treatment was 0.027–0.183 m and in the high wave exposure treatment was 0.04–0.261 m (see Experiment 2 methods for details). Stormy weather during the experiment produced particularly high wave exposure at La Vela (S3 Fig.) making conditions at the site a good proxy for future projected increases [21].

The sediment load was reduced by gently waving water across the tile for 5 seconds to resuspend the deposited material and ensuring that no visible sediment remained [39, 40]. This treatment was applied every 7–10 days, and lead to an overall reduction of sediment by 20% (± 5%) throughout the experimental period, at both the sheltered and exposed positions. These manipulations were designed to reduce the sediment to levels measured within dense healthy stands of *C*. *barbata* in eastern Adriatic Sea and elsewhere in the Mediterranean Sea (S1 Table).

At the conclusion of the experiment, mid July 2013, we counted the number of recruits and measured their maximum height on each tile (to the nearest mm) using a micrometre (10× magnification). The effects of the sediment load (2 fixed levels = ambient and reduced) and wave exposure (2 fixed levels = low and high) on the number and length of the recruits on each tile (n = 4) were analysed with 2-way ANOVAs. For all analyses, data were checked for both normality (using normal probability plots) and homoscedasticity. Boxcox plots were used to determine the appropriate transformation to stabilise variances.

### Experiment 2: Effects of reducing the sediment load at different levels of wave exposure on juveniles in tanks

The *C*. *barbata* juveniles used in Experiments 2 and 3 were collected from random positions at La Vela in June and July, respectively, after the recruitment period. Boulders at La Vela were broken into small fragments (~200–700 mm^2^), which contained an average of 3 and a maximum of 6 individuals. This technique for collecting and transplanting juveniles has been used successfully to conduct other experiments with no detectable impacts on algal growth or survival relative to unmanipulated controls [21]. All juveniles were left to acclimate for 4 weeks in the tanks or mesocosms before commencing the experiments.

In late August 2012, we ran a 4 month laboratory experiment to test the effects of reducing the sediment load at different levels of wave exposure on juveniles. The experiment was conducted in 4 large tanks (120 × 100 × 74 cm; 346 litres) located in Yerseke, Netherlands. The juveniles were transported to Yerseke in polyethylene bottles (5 litres) filled with site seawater in controlled temperature conditions that were similar to the Adriatic Sea. Individual rock fragments with juveniles were attached to 32 ceramic bricks (24 × 12 × 5 cm) using Silicone glue (Den Braven) and placed randomly ~400 mm apart in the tanks. There were 8 bricks in each tank. The sediment treatments were applied directly to the bricks and the wave treatments to the tanks however, the effects of the waves varied throughout the tank. Because of the experiment constraints there was only 1 tank per treatment. However, at 1.5 and 3 months each group of 8 bricks was moved to another tank and position within the tank (allocated randomly) and the relevant sediment and wave treatments were re-applied to each brick. Although it was not possible to quantify and explicitly test the potential effect of the tank using this method, any variability related to this factor is correctly spread through the different treatments. As the treatments were applied to the bricks, we considered each brick as independent replicate for the experiment (resulting in n = 8 replicate bricks for each sediment × wave treatment).

The tanks were filled with filtered seawater from the Eastern Scheldt estuary, which was changed weekly. Light was supplied by 5× GreenPower LED lights and the average surface irradiance was 50 μmol m^−2^ s^−1^ (SE = ± 7.5), and the photoperiod was set at 12:12 hours light: dark, which was similar to conditions at La Vela in 2012 during the *C*. *barbata* growth season (derived from n = 8 measurements per month). The tanks were kept in a controlled temperature room at 24°C to mimic field conditions and continuously aerated using compressed air.

We applied 2 wave treatments: low (no water motion) and high (0.16–0.33 m relative wave height measured using a Druck PTX 105 1830 pressure sensor), which correspond to future predictions of wave exposure at La Vela [21] (S3 Fig.). We downloaded wave data collected at a buoy located ~130 km north of La Vela (44.2155°N, 12.4766°E), during 2008–2012 (S3 Fig.). We also deployed a wave gauge OSSI-010–003C (Ocean Sensor Systems) at La Vela at a depth of 4 m, for 10 days in September 2012. We compared the data from the wave gauge and buoy during the same period. There was a significant agreement in the wave heights (Willmott = 0.9191), thus wave data from the buoy was used as approximation of wave exposure at La Vela (S3 Fig.). The waves in the tanks were generated using 2 hydraulic wave 93 generators which operated 24 h daily [41].

The sediment treatments were ambient (3000 g m^−2^) and reduced (750 g m^−2^). Ambient levels matched the field treatments in Experiment 1 and were derived directly from measurements taken at La Vela [34], while reduced levels were average levels measured in healthy *C*. *barbata* forests in eastern Adriatic Sea and elsewhere in the Mediterranean (S1 Table). Both treatment levels (ambient and reduced) were within those measured at the site (S1 Fig.). The sediment (3/4 fine and 1/4 coarse) was applied as a fine rain [42].

At the beginning of the experiment and after 4 months we counted the number of juveniles (3–6 individuals) on each brick and measured their maximum height (from base to tip) using callipers. At the conclusion of the experiment, December 2012, we calculated the percentage survival and average relative change in height of the juveniles on each brick. All stipes were shaved off the rock fragments on the bricks, their filamentous epiphytic algae removed, oven dried at 80°C for 7 days and then weighed to the nearest 0.001 g. We also measured the average photosynthetic stress as the relative electron transport yield (F_v_F_m_ ratio) of the juveniles on the bricks using a Water-PAM (pulse amplitude modulated florometer) (Walz, Germany). All stipes were dark adapted for 15 minutes, placed in a darkened Petri dish and then measurements were taken in the middle of the stipe, using a FIBER optic probe.

The effects of the sediment load (2 levels = ambient and reduced) and wave exposure (2 levels = low and high) on the survival, and average growth, dry weight, and photosynthetic stress of the juveniles and the dry weight of the filamentous epiphytic algae for each brick (n = 8) were tested using 2-way ANOVAs.

### Experiment 3: Effects of reducing nutrient concentrations and the sediment load at different levels of temperature on juveniles in mesocosms

In late June 2012, we undertook a 4 month laboratory experiment to test the effects of reducing nutrient concentrations and the sediment load at different levels of temperature on *C*. *barbata* juveniles. For this experiment we could not run the equivalent test in the field at the recruitment stage (as we did for Experiment 1) because of the difficulties in reducing nutrient concentrations and increasing temperature *in situ*. The experiment was undertaken in 32 mesocosms located in Ravenna Italy. Individual rock fragments with 3–6 juveniles (see above section for details) were placed directly into each mesocosm. All nutrient, sediment and temperature treatments were randomly applied to the mesocosms (resulting in n = 4 replicate mesocosms for each nutrient × sediment × temperature treatment).

Each mesocosm (24.5 × 18 × 13.5 cm) contained 2.2 litres and had a continuous flow-through of sterilized seawater collected from the Marche Region at 250 litres per day from 4 reserve tanks. The light was supplied by 4 Paulman florescent lamps of 36 watts, and the surface irradiance and the photoperiod were set to the same conditions as in Experiment 2. The seawater in the reserve tanks was continuously aerated using FERPLAST BluePower pumps.

The temperatures were average current (24°C) (S4 Fig.) and projected future (27°C). The increased temperature was based on predictions from the IPCC model scenario A2 for 2100 which has been applied to the Mediterranean Sea [43]. All mesocosms were placed in a control temperature room which was set at 24°C. We used TetraTec HT 150 w heaters to raise the temperature to 27°C.

The nutrients treatments were ambient (DIN = 15 μmol l^−1^, PO_4_ = 0.15 μmol l^−1^ [28] (S1 Fig.) and reduced (DIN < 6.5 μmol l^−1^, PO_4_ < 0.05 μmol l^−1^). The reduced nutrient levels chosen for the experiment were based recommendations by the European Environmental Agency (www.eea.europa.eu/publications/ENVIASSRP04) and on the lower levels observed in the study site region (S2 Fig.). For the reduced nutrient treatment we collected seawater for the experiment in an area1000 m offshore with low nutrient concentrations. The ambient nutrient treatment was achieved by adding 0.25 g of slow release Plantacote 6 mol N:P:K fertilizer to raise nutrients in the seawater in the mesocosms to the same levels as those to which *C*. *barbata* was naturally subjected in the field [28] (S2 Fig.). The Plantacote pellets were supplied in mesh bags that were changed every two weeks. We monitored the nutrient concentrations in the mesocosms by randomly sampling the water in 2 mesocosms from 8 possible choices (nutrient × temperature, irrespectively of sediment), monthly.

The amount of sediment added (3000 g m^−2^ and 750 g m^−2^) and timings (0, 1.5 and 3 months), were the same as in Experiment 2 (see above for full details). We removed all of the remaining sediment before the new treatments were applied.

At the beginning of the experiment and after 4 months we measured the percentage survival, relative change in height and weight of the juveniles and weight of filamentous epiphytic algae as described above in Experiment 2 methods.

The effects of the sediment load (2 fixed levels = ambient and reduced), nutrient concentration (2 fixed levels = ambient and reduced) and temperature (2 levels = ambient and increased) on the number, average growth, and dry weight of the juveniles and the dry weight of the filamentous epiphytic algae in each mesocosms (n = 4) were tested using 3-way ANOVAs. The effects of adding fertilizer (2 levels = ambient and reduced) and manipulating the temperature (2 levels = ambient and increased) on the nutrient concentrations in mesocosm seawater (n = 2) were also tested using 2-way ANOVAs.

### Comparisons between laboratory and field controls

To verify how closely our laboratory treatments matched the field conditions, we monitored the responses of juveniles at the field site (n = 4) throughout their growth season. We attached individual rock fragments with juveniles (3–6 individuals) to marble tiles on boulders at La Vela in late June (juveniles collected for Experiment 3) and July (juveniles collected for Experiment 2), at sheltered positions in 2.5–3.5 m in depth using epoxy putty. The differences in the survival, growth, and dry weight of the juveniles and dry weight of the filamentous epiphytic algae between the field controls and the ambient treatments in Experiment 2 and 3 were analysed using t-tests.

### Comparison of local management options

We examined the effects of different management scenarios (reduced vs ambient) of local stressors (sediment and/or nutrients) on the percentage survival of *C*. *barbata* with projected increased in climate stressors (high wave exposure or increased temperature) using the log ratio effect size:

Experiment 1—juveniles of *C*. *barbata*: (log (reduced sediment at high wave exposure/ambient sediment at high wave exposure)*100)

Experiment 2—recruits of *C*. *barbata*: (log (reduced sediment at high wave exposure/ambient sediment at high wave exposure)*100)

Experiment 3—juveniles of *C*. *barbata*: (log (reduced sediment, reduced nutrients or reduced sediment and nutrients at increased temperature/ambient sediment, ambient nutrients or ambient sediment and nutrients at increased temperature)*100)

## Results

### In situ measurements of sediment and nutrients

The level of sediment in the ambient treatments for Experiments 2 and 3 were similar to the natural levels measured at La Vela in 2012 (S1 Fig.). Similarly, throughout April—September 2012 there were no detectable differences between the ambient nutrient concentrations in Experiment 3 and those measured at La Vela (t = -2.149, P > 0.05, S1 Fig.). The level of sediment [34] (S1 Table) and nutrient concentrations [28] (S2 Fig.) at La Vela in 2012 were also similar to those recorded in the Monte Conero region between 2008 and 2011.

### Experiment 1: Effects of reducing the sediment load at different levels of wave exposure on recruits *in situ*

**Fig 1:**
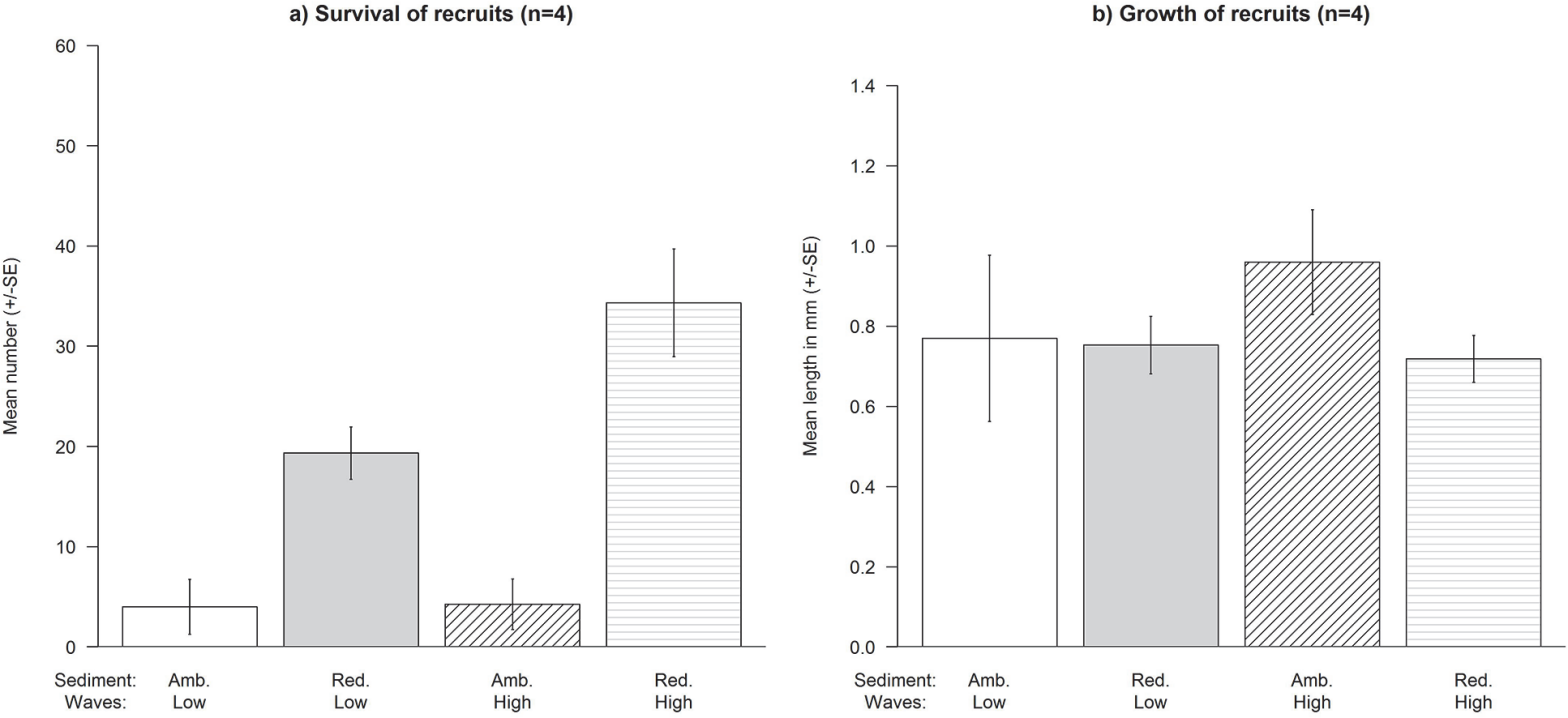
Effects of reducing the sediment load at different levels of wave exposure on the mean (± SE) of: a) number and the b) length of recruits on tiles (1000 cm^2^) in Experiment 1. Treatments are: ambient sediment, low wave exposure; reduced sediment, high wave exposure; ambient sediment, low wave exposure; and reduced sediment, high wave exposure.

At the current ambient sediment load the density of *C*. *barbata* recruits was extremely low (≤ 12 individuals per 1000 cm^2^) (Fig. 1). Reducing the sediment load significantly enhanced the number of recruits (Fig. 1), particularly at high wave exposure (Sediment × Waves: F_1,12_ = 5.906, P = 0.0411, Fig. 1). In contrast, there were no detectable effects of reducing sediment (Sediment: F_1,12_ = 0.161, P > 0.05, Fig. 1) or of high wave exposure (Waves: F_1,12_ = 0.161, P > 0.05, Fig. 1) on the average length of the recruits.

### Experiment 2: Effects of reducing the sediment load at different levels of wave exposure on juveniles in tanks

**Fig 2:**
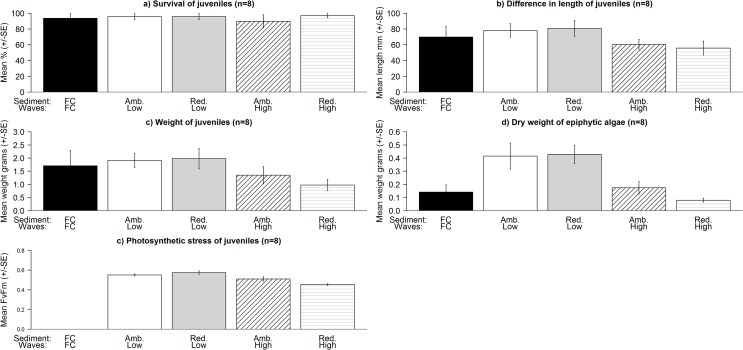
Effects of reducing the sediment load at different levels of wave exposure on the mean (± SE): a) survival (%), b) average difference in growth rate (mm), c) dry weight (g) and d) dry weight of epiphytic algae (g) and e) the photosynthetic stress (F_v_F_m_) of juveniles (3–6 individuals) on rock fragments (~200–700 mm^2^) on bricks in Experiment 2. Treatments are: field control (FC); ambient sediment, low wave exposure; reduced sediment, low wave exposure; ambient sediment, high wave exposure; and reduced sediment, high wave exposure. There were no measurements of photosynthetic stress taken for the field controls.

At high wave exposure, there were significant declines in the growth (Waves: F_1,32_ = 3.014, P = 0.048), dry weight (Waves: F_1,32_ = 3.253, P = 0.042) and photosynthetic stress (Waves: F_1,32_ = 28.275, P < 0.001) of juveniles and in the dry weight of filamentous epiphytic algae on juveniles (Waves: F_1,32_ = 13.275, P < 0.001) (Fig. 2). However, there were no detectable effects of reducing the current sediment load on any of the parameters measured for *C*. *barbata* juveniles (survival; Sediment: F_1,32_ = 0.433, P > 0.05, growth: F_1,32_ = 0.042, P > 0.05, dry weight; Sediment: F_1,32_ = 0.305, P > 0.05, photosynthetic stress, Sediment: F_1,32_ = 0.436, P > 0.05 and dry weight of epiphytic algae; Sediment: F_1,32_ = 0.46, P > 0.05, Fig. 2).

### Experiment 3: Effects of reducing nutrient concentrations and the sediment load at different levels of temperature on juveniles in mesocosms

**Fig 3:**
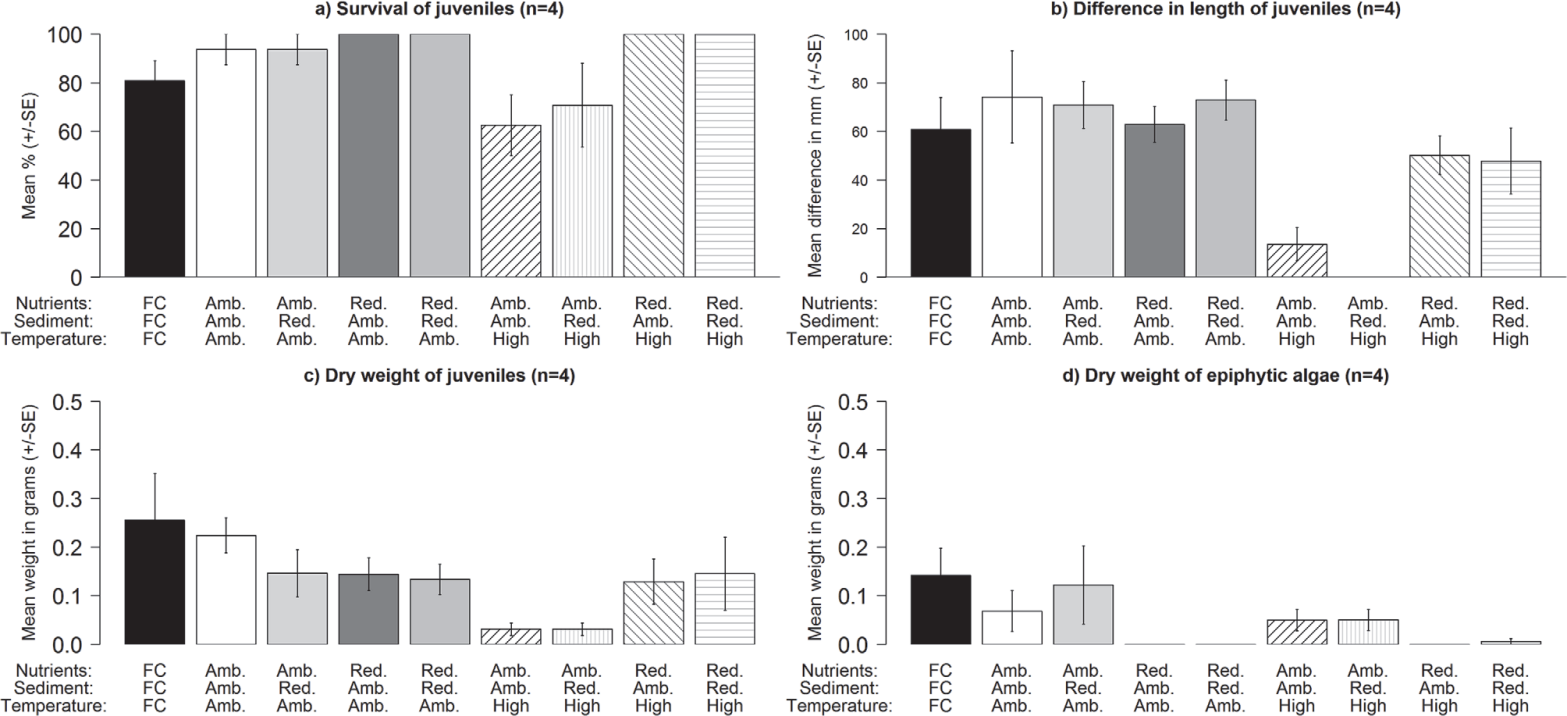
Effects of reducing nutrient concentrations and the sediment load at different levels of temperature on the mean (± SE): a) survival (%), b) average difference in growth rate (mm), c) dry weight (g) and d) dry weight of epiphytic algae (g) of juveniles (3–6 individuals) on rock fragments (~200–700 mm^2^) in mesocosms in Experiment 3. Treatments are: field control (FC);ambient nutrients, ambient sediment, ambient temperature; ambient nutrients, managed sediment, ambient temperature; reduced nutrients, ambient sediment, ambient temperature; reduced nutrients, reduced sediment, ambient temperature; ambient nutrients, ambient sediment, high temperature; ambient nutrients, reduced sediment, high temperature; reduced nutrients, ambient sediment, high temperature; and reduced nutrients, reduced sediment, high temperature.

At increased temperature, reducing ambient nutrient concentrations resulted in increased survival (Nutrients × Temperature: F_1,32_ = 5.53, P = 0.026), growth (Nutrients × Temperature: F_1,32_ = 10.06, P = 0.004) and dry weight (Nutrients × Temperature: F_1,32_ = 4.517, P = 0.043) of *C*. *barbata* juveniles (Fig. 3). Reducing the sediment load and the sediment load and nutrient concentrations combined had no added benefits for juveniles (Fig. 3). In contrast, reducing ambient nutrient concentrations, resulted in declines in the dry weight of filamentous epiphytic algae on juveniles (Nutrients: F_1,32_ = 7.224, P > 0.05, Fig. 3), irrespective of the sediment load or the temperature.

Throughout the experiment, there was a significantly higher concentrations of DIN (F_2,35_ = 7.957, P = 0.001) and PO_4_ (F_2,35_ = 14.427, P < 0.001) in the ambient nutrient treatments relative to the reduced nutrient treatments (S1 Fig.).

### Comparisons between laboratory and field controls

At the conclusion of the laboratory experiments, the survival (Experiment 2: t = 0.277, P > 0.05, Experiment 3: t = 1.252, P > 0.05), growth (Experiment 2: t = 0.522, P > 0.05, Experiment 3: t = 0.578, P > 0.05) and dry weight (t = 0.319, P > 0.05, Experiment 3: t = -0.304, P > 0.05) of *C*. *barbata* juveniles in the Experiment 3 and 2 treatments simulating ambient conditions were similar to that of the field controls, deployed in late June (for Experiment 3) and July 2012 (for Experiment 2) (Figs. 2 and 3). There was, however, a significantly higher dry weight of epiphytic filamentous algae on the juveniles in the tanks in Experiment 2 (t = 2.399, P = 0.038) but not on the juveniles in the mesocosms in Experiment 3 (t = -1.046, P > 0.05) relative to the field controls (Figs. 2 and 3).

### Comparing local management options

**Fig 4:**
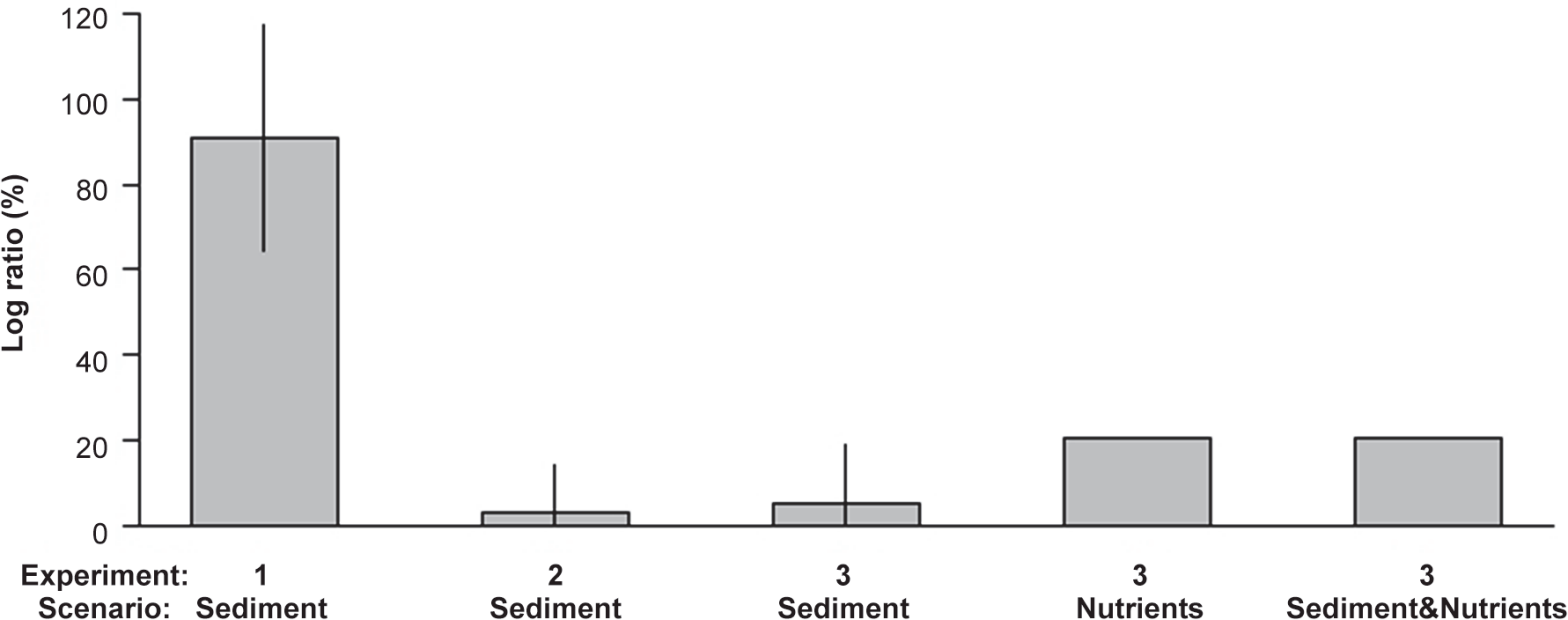
Effects of different management scenarios relative to current ambient conditions on the survival of early life stages of *C*. *barbata* with projected increases in climatic stressors. Scenarios are: Experiment 1: Effects of reducing the sediment load at high wave exposure on recruits, Experiment 2: Effects of reducing the sediment load at high wave exposure on juveniles, Experiment 3: Effects of reducing the sediment load, reducing nutrient concentrations and reducing the sediment load and nutrient concentrations at increased temperature on juveniles. Bars are the mean log ratio (± SE) percentage increase for each experiment.

Relative to the current levels, reducing the sediment load resulted in a 90.24% increase in the density of *C*. *barbata* recruits at high wave exposure (Fig. 4, Experiment 1) but there very little benefits for the juveniles (Fig. 4, Experiment 2). Reducing ambient nutrient concentrations enhanced the survival of juveniles by 20.14% with projected increases in temperature (Fig. 4, Experiment 3) but there were no added benefits of reducing nutrient concentrations and the sediment load in combination (Fig. 4, Experiment 3) and no detectable improvements when reducing the sediment load alone (Fig. 4, Experiment 3).

## Discussion

An increasing number of studies have demonstrated that the interactions between local anthropogenic and global climatic stressors can lead to dramatic loss of key habitats [3, 4, 5, 11, 23, 24]. However, to date there has very little experimental research on whether reducing levels of local anthropogenic stressors can be used to mitigate or prevent their decline [7, 12, 20]. Using *C*. *barbata* as a model keystone species we demonstrated that the reduction of local anthropogenic stressors could enhance the resilience of these canopy algae to global climatic threats. Reducing the sediment load increased the density of *C*. *barbata* recruits, particularly at high wave exposure, and decreasing nutrient concentrations resulted in greater survival and growth of *C*. *barbata* juveniles at increased temperature.

High sediment load is well known for its detrimental effects on the settlement and recruitment stages of canopy algae [25]. Even a relatively thin layer of sediment can inhibit the establishment and survival of many canopy species [30, 34, 37, 44]. Sedimentation has been suggested to be one of the main factors triggering the shifts from canopy to turf-forming algae [33]. The effects of sediment can be amplified or altered by interacting with other stressors [25]. Reducing the ambient sediment load was very effective at enhancing the density of *C*. *barbata* recruits, particularly at high wave exposure by reducing the effects of abrasion and scour [36, 37]. Conversely, reducing the sediment load had little benefits for the later life stages of *C*. *barbata* (i.e. juveniles), which are likely to be more tolerant to the effects of this stressor [30, 34]. These findings suggest that effective management requires not only knowledge of the relevant multiple stressors acting in a system and their possible interactions, but also of their impacts on the various life stages of the key species.

For *C*. *barbata* juveniles, reducing nutrient concentrations appeared to be the most effective factor enhancing their resilience to high temperature. Although some studies have reported the interactions between high nutrients concentrations and increased temperature can have negative effects on the growth and survival of canopy algae [45, 46, 47] the underlying mechanisms remain unclear. We hypothesise these results might be explained by the effects of these stressors on the microbial community [48]. Bacterial growth is enhanced in eutrophic marine systems [49] which could have negative feedbacks on the host species of canopy algae. Such effects could be elevated at high temperature, which can reduce the levels of chemical defences on the host thalli leading to colonization or proliferation by opportunistic pathogens [50, 51].

Overall our results suggest the reducing the sediment load is the most important factor in increasing the resilience of *C*. *barbata* populations to project future global climatic stressors by significantly enhancing the density of recruits, while the reduction of nutrient concentrations would also provide some further benefits for juveniles. The implications are that the reduction of local stressors could be equally as effective at disrupting the negative effects of additive interactions (e.g. high sediment × high wave exposure) as non-additive interactions (e.g. high nutrients × increased temperature) for canopy-forming algae. Thus, developing effective management strategies for canopy-forming algae requires detailed knowledge of the effects of adding and reducing local stressors on the interactions between local and global stressors [5, 6, 7].

Tank-based experiments provide important insights into the effects of stressors that are difficult to manipulate in the field (e.g., increased temperature and wave exposure and reduced nutrient concentrations and sediment load) [7]. However, it is important to check how well our laboratory-based experiments mimic the site conditions of interest. The sediment load, nutrient concentrations and the growth and survival of algae in our laboratory experiments (Experiments 2 and 3) were statistically similar to those measured at the study site. The filamentous epiphytic algae on the juveniles in the tanks in Experiment 2 were higher than in the field, which could be linked to prolonged exposure to low flow conditions, but did not appear to affect the growth or density of the juveniles. This implies that the conditions in the laboratory and the responses of the algae were likely to be representative of those found in the north western Adriatic Sea. Our future scenarios were also within the range of current conditions experienced by *C*. *barbata* at the field site, throughout their recruitment and growth season. Thus, implementing management options for the current sediment load and nutrients concentrations are likely to be important for enhancing and protecting current populations of *C*. *barbata* and will be vital for ensuring the resilience of these foundation species, even under the most optimistic of future global climate change scenarios.

We highlight the importance of understanding the nature and effects of stressor interactions on key habitat forming species. To date, management strategies have typically advocated the reduction of all stressors (whether local or global-scale) to ensure the resilience of desirable ecosystems configurations [8, 52]. However, with current delays and limitations on the mitigation of global stressors, it is fundamental to identify alternative approaches that can work at local scales [6]. We clearly show that reducing common local anthropogenic stressors can enhance the resilience of key habitat forming species to global stressors. Determining what is the most important local stressor for remediation depends on identifying the specific global threat(s) in the system (i.e. increasing wave exposure, temperature or both). Indeed, our results show that, for some systems management actions for individual local stressors may be more important than trying to reduce all of the local stressors or remediate the global stressors.

Improving water quality (reducing the sediment load and nutrient concentrations) could be an effective local intervention to prevent habitat shifts from canopy to mat-forming algae, in enclosed seas, bays and estuaries [5, 7, 33]. The European Commission has developed several recommendations for monitoring and controlling nutrient concentrations in coastal waters through improved agriculture techniques and shifts to organic fertilizers or farming techniques, however currently there are no guidelines for *in situ* testing or management of sediment inputs (http://ec.europa.eu). The potential initiatives for reducing sediment inputs include limiting or reducing beach nourishments and dredging (particularly throughout the *C*. *barbata* recruitment period), bank revegetation and stabilisation and installing traps in nearby river catchment areas [25]. All of these policies are costly, requiring the co-ordination and collaboration of coastal managers, landowners, stakeholders, local business and other interested parties, and trade-offs in land use to achieve effective results at large-scales. Thus, identifying the maximum levels of sediment and nutrients at which populations of canopy algae can still persist remains a critical next step for prioritising management actions.

## Supporting Information

S1 Fig Temporal variability in the mean (± SE): a) concentration of DIN (μmol l-1), b) concentration of PO_4_ (μmol l-1), at La Vela and in the mesocosms in Experiment 3 and c) amount of sediment deposited (g m^−2^ d^−1^), (n = 2), and d) amount of sediment accumulated (g m^−2^) (n = 8) at Monte Conero between April and October 2012. The sediment [34] (S1 Table) and nutrient [28] (S2 Fig.) data collected at the site were compared with other data from Monte Conero region collected between 2008 and 2011. Gaps are months in which the data was not collected. Codes are: FC = field control; aNaT = ambient nutrients, ambient temperature; rNaT = reduced nutrients, ambient temperature; aNhT = ambient nutrients, high temperature; rNhT = reduced nutrients, high temperature.
(TIF)

S2 Fig Monthly variability the mean (± SE) a) concentration of DIN (μmol l-1), b) concentration of PO_4_ (μmol l-1) at Monte Conero between 2008 and 2011. Gaps are missing data. Data was obtained from ARPA Marche, courtesy of G. De Grandis.
(TIF)

S3 Fig Monthly variability the a) average, b) maximum and c) minimum wave height (m) measured at buoy located in 10 m depth at Cesenatico between 2007 and 2012. Data was downloaded from ARPA Emilia Romagna via http://dexter-smr.arpa.emr.it/. Gaps are missing data.
(TIF)

S4 Fig Monthly variability the average (± SE) sea surface temperature measured at a buoy located at Ancona between 2000 and 2012. Data was obtained from ISPRA, courtesy of Dr G. Sara. Gaps are missing data.
(TIF)

S1 Table Mean and maximum (± SE) amounts of sediment accumulation and deposited in degraded *C*. *barbata* forests in Monte Conero and healthy forests in Corsica and Rovinj. Measurements were taken in summer of 2009. Data obtained courtesy of Dr S. Perkol-Finkel.
(DOCX)

## References

[1] Airoldi L, Beck M. Loss, status and trends for coastal marine habitats of Europe. Oceanogr Mar Biol Annu Rev 2007; 45: 345–405.

[2] Lotze HK, Lenihan HS, Bourque BJ, Bradbury RH, Cooke RG, et al Depletion, degradation, and recovery potential of estuaries and coastal seas. Science 2006; 312: 1806–1809. 16794081 10.1126/science.1128035

[3] Ban SS, Graham NAJ, Connolly SR. Evidence for multiple stressor interactions and effects on coral reefs. Glob Chang Biol 2014; 20: 681–697. 24166756 10.1111/gcb.12453

[4] Crain C, Kroeker K, Halpern B. Interactive and cumulative effects of multiple human stressors in marine systems. Ecol Lett 2008; 11: 1304–1315. 10.1111/j.1461-0248.2008.01253.x 19046359

[5] Strain EMA, Thomson RJ, Micheli F, Mancuso FP, Airoldi L. Identifying the interacting roles of stressors in driving the global loss of canopy-forming algae to mat-forming algae in marine ecosystems. Glob Chang Biol 2014; 20: 3300–3312. 10.1111/gcb.12619 24771500

[6] Brown CJ, Saunders MI, Possingham HP, Richardson AJ. Managing for interactions between local and global stressors of ecosystems. PLoS ONE 2013; 8: e65765 10.1371/journal.pone.0065765 23776542 PMC3680442

[7] Falkenberg LJ, Connell SD, Russell BD. Disrupting the effects of synergies between stressors: improved water quality dampens the effects of future CO_2_ on a marine habitat. J Appl Ecol 2013; 50: 51–58.

[8] Cote IM, Darling ES. Rethinking ecosystem resilience in the face of climate change. PLoS Biology 2010; 8: e1000438 10.1371/journal.pbio.1000438 20668536 PMC2910654

[9] Russell BD, Connell SD. Origins and consequences of global and local stressors: incorporating climatic and non-climatic phenomena that buffer or accelerate ecological change. Mar Biol 2012; 159: 2633–2639.

[10] Darling ES, Cote IM. Quantifying the evidence for ecological synergies. Ecol Lett 2008; 11: 1278–1286. 10.1111/j.1461-0248.2008.01243.x 18785986

[11] Wahl M, Jormalainen V, Eriksson BK, Dethier M, Karez R, et al Stress ecology in *Fucus*: abiotic, biotic and genetic interactions. Adv Mar Biol 2011; 59: 37–105. 10.1016/B978-0-12-385536-7.00002-9 21724018

[12] Carilli JE, Norris RD, Black BA, Walsh SM, Mc Field M. Local stressors reduce coral resilience to bleaching. PLoS ONE 2009; 4: e6324 10.1371/journal.pone.0006324 19623250 PMC2708352

[13] Steneck RS, Graham MH, Bourque BJ, Corbett D, Erlandson JM, et al Kelp forest ecosystems: biodiversity, stability, resilience and future. Environ Conserv 2002; 29: 436–459.

[14] Schiel D, Foster MS. The population biology of large brown seaweeds: ecological consequences of multiphase life histories in dynamic coastal environments. Ann Rev Ecol Syst 2006; 37: 343–372.

[15] Benedetti-Cecchi L, Pannacciulli P, Bulleri F, Moschella PS, Airoldi L, et al Predicting the consequences of anthropogenic disturbance: large-scale effects of loss of canopy algae on rocky shores. Mar Ecol Prog Ser 2001; 214: 137–150.

[16] Connell SD, Russell BD, Turner DJ, Shepherd SA, Kildea T, et al Recovering a lost baseline: missing kelp forests from a metropolitan coast. Mar Ecol Prog Ser 2008; 360: 63–72.

[17] Foster MS, Schiel DR. Loss of predators and the collapse of southern California kelp forests (?): Alternatives, explanations and generalizations. J Exp Mar Biol Ecol 2010; 393: 59–70.

[18] Connell SD, Foster MS, Airoldi L. What are algal turfs? Towards a better description of turfs. Mar Ecol Prog Ser 2014; 495: 299–307.

[19] Bianchi CN, Morri C, Chiantore M, Montefalcone M, Parravicini V, et al Mediterranean sea biodiversity between the legacy from the past and a future of change In: Stambler N, editor. Life in the Mediterranean Sea: A look at habitat change. New York: Nova Science Publishers, Inc 2012 pp. 1–55.

[20] Falkenberg LJ, Russell BD, Connell SD. Stability of strong species interactions resist the synergistic effects of local and global pollution in kelp forests. PLoS ONE 2012; 7: e33841 10.1371/journal.pone.0033841 22439005 PMC3306304

[21] Perkol-Finkel S, Airoldi L. Loss and recovery potential of marine habitats: an experimental study of the factors maintaining the resilience in subtidal algal forests at the Adriatic Sea. PLoS ONE 2010; 5: e10791 10.1371/journal.pone.0010791 20520726 PMC2875393

[22] Airoldi L, Ballesteros E, Buonuomo R, van Belzen J, Bouma TJ, et al. Marine forests at risk: solutions to halt the loss and promote the recovery of Mediterranean canopy-forming seaweeds. Proceedings of the 5th Mediterranean Symposium on Marine vegetation. Portoroz, Slovenia. 2014. pp. 13.

[23] Parravicini V, Micheli F, Montefalcone M, Morri C, Villa E, et al Conserving biodiversity in a human-dominated world: degradation of marine sessile communities within a protected area with conflicting human uses. PLoS ONE 2014; 8: e75767. 10.1371/journal.pone.0075767 PMC3797118 24143173

[24] Bianchi CN, Corsini-Foka M, Morri C, Zenetos A. Thirty years after: dramatic change in the coastal marine ecosystems of Kos Island (Greece), 1981–2013. Mediterr Mar Sci 2014; 15: 482–497.

[25] Airoldi L. The effects of sedimentation on rocky coast assemblages. Oceanogr Mar Biol Annu Rev 2003; 41: 161–236.

[26] Munda IM, Veber M. Simultaneous effects of trace metals and excess nutrients on the Adriatic seaweed *Fucus virsoides* (Don.) J. Ag. (Phaeophyceae, Fucales). Botanica Marina 1996, 39: 297–309.

[27] Nikolic V, Zuljevic A, Mangialajo L, Antolic B, Kuspilic G, et al Cartography of littoral rocky-shore communities (CARLIT) as a tool for ecological quality assessment of coastal waters in the Eastern Adriatic Sea. Ecol Indic 2013; 34: 87–93.

[28] Accorroni S, Romagnoli T, Colombo F, Pennesi C, Di Camillo CG, et al *Ostreopsis* cf. *ovata* bloom in the northern Adriatic Sea during summer 2009: Ecology, molecular characterization and toxin profile. Mar Poll Bull 2011; 62: 2512–2519. 10.1016/j.marpolbul.2011.08.003 21903227

[29] Bergstrom L, Berger R, Kautsky L. Negative direct effects of nutrient enrichment on the establishment of *Fucus vesiculosus* in the Baltic Sea. Eur J Phycol 2003; 38: 41–46.

[30] Eriksson BK, Johansson G. Effects of sedimentation on macroalgae: species-specific responses are related to reproductive traits. Oecologia 2005; 143: 438–448. 15682344 10.1007/s00442-004-1810-1

[31] Krause-Jensen D, Sagert S, Schubert H, Bostrom C. Empirical relationships linking distribution and abundance of marine vegetation to eutrophication. Ecol Indic 2008; 8: 515–529.

[32] Coleman MA, Kelaher BP, Steinberg PD, Millar AJK. Absence of a large brown macroalga on urbanized rocky reefs around Sydney, Australia and evidence for historical decline. J Phycol 2008; 44: 897–901. 27041607 10.1111/j.1529-8817.2008.00541.x

[33] Gorman D, Connell SD. Recovering subtidal forests in a human-dominated landscape. J App Ecol 2009; 46: 1258–1265.

[34] Irving AD, Balata D, Colosio F, Ferrando GA, Airoldi L. Light, sediment, temperature, and the early life-history of the habitat-forming alga *Cystoseira barbata* . Mar Biol 2009; 156: 1223–1231.

[35] Sala E, Ballesteros E, Dendrinos P, Di Franco A, Ferretti F, et al The structure of Mediterranean rocky reef ecosystems across environmental and human gradients, and conservation implications. PLoS ONE 2012; 7: e32742 10.1371/journal.pone.0032742 22393445 PMC3290621

[36] Araujo R, Arenas F, Faberg P, Sousa-Pinto I, Serrao EA. The role of disturbance in differential regulation of co-occurring brown algae species: Interactive effects of sediment deposition, abrasion and grazing on algae recruits. J Exp Mar Biol Ecol 2012; 422: 1–8.

[37] Devinny JS, Volse LA. Effects of sediments on the development of *Macrocystis pyrifera* gametophytes. Mar Biol 1978; 48: 343–348.

[38] RAC/SPA. Action plan for the conservation of marine vegetation in the Mediterranean Sea. 2006. 51 pp.

[39] Connell SD. Assembly and maintenance of subtidal habitat heterogeneity: synergistic effects of light penetration and sedimentation. Mar Ecol Prog Ser 2005; 289: 53–61.

[40] Irving AD, Connell SD. Sedimentation and light penetration interaction to maintain heterogeneity of subtidal habitats: algal versus invertebrate dominated assemblages. Mar Ecol Prog Ser 2002; 245: 83–91.

[41] La Nafie YA, de los Santos CB, Brun FG, van Katwijk MM, Bouma TJ. Waves and high nutrient loads jointly decrease survival and separately affect morphological properties in the seagrass *Zostera noltii* . Limnol Oceanogr 2012; 57: 1664–1672.

[42] Airoldi L, Virgilio M. Responses of turf-forming algae to spatial variations in the deposition of sediments. Mar Ecol Prog Ser 1998; 165: 271–282.

[43] Somot S, Sevault F, Deque M, Crepon M. 21st century climate change scenario for the Mediterranean using a coupled atmosphere ocean regional climate model. Glob Planet Change 2008; 63: 112–126.

[44] Deiman M, Iken K, Konar B. Susceptibility of *Nereocystis luetkeana* (Laminariales, Ochrophyta) and *Eualaria fistulosa* (Laminariales, Ochrophyta) spores to sedimentation. Algae 2012; 27: 115–123.

[45] da Costa Braga A, Valentin YY. Growth of *Laminaria abyssalis* (Phaeophyta) at different nitrate concentrations. Phycologia 1994; 33: 271–274.

[46] Kremer BP, Munda IM. Ecophysiological studies of the Adriatic seaweed, *Fucus virsoides* . Mar Ecol 1982; 3: 75–93.

[47] Yarish C, Penninman CA, Egan B. Growth and reproductive responses of *Laminaria longucruris* (Laminariales, Phaeophyta) to nutrient enrichment. Hydrobiologia 1990; 284: 505–511.

[48] Singh RP, Reddy CRK. Seaweed-microbial interactions: key functions of seaweed-associated bacteria. FEMS Microbiol Ecol 2014; 88: 213–230. 10.1111/1574-6941.12297 24512602

[49] Xu J, Kong JH, Harrison PJ, Liu H. Effect of seawater-sewage cross-transplants on bacterial metabolism and diversity. Microb Ecol 2013; 66: 60–72. 10.1007/s00248-013-0207-2 23494574

[50] Campbell AH, Harder T, Nielsen S, Kjelleberg S, Steinberg PD. Climate change and disease: bleaching of a chemically defended seaweed. Glob Chan Biol 2011; 17: 2958–2970.

[51] Fernandes N, Steinberg PD, Rusch D, Kjelleberg S, Thomas T. Community structure and functional gene profile of bacteria on healthy and diseased thalli of the red seaweed *Delisea pulchra* . PLos ONE 2012; 7: e50854 10.1371/journal.pone.0050854 23226544 PMC3513314

[52] Lotze HK, Coll M, Magera AM, Ward-Paige C, Airoldi L. Recovery of marine animal populations and ecosystems. Trends Ecol Evol 2011; 26: 595–605. 10.1016/j.tree.2011.07.008 21852017

