# Supplementary figures and images for: Management of Local Stressors Can Improve the Resilience of Marine Canopy Algae to Global Stressors
Source: PLoS One. 2015 Mar 25;10(3):e0120837. doi: 10.1371/journal.pone.0120837 (PMC4373769; doi:10.1371/journal.pone.0120837)

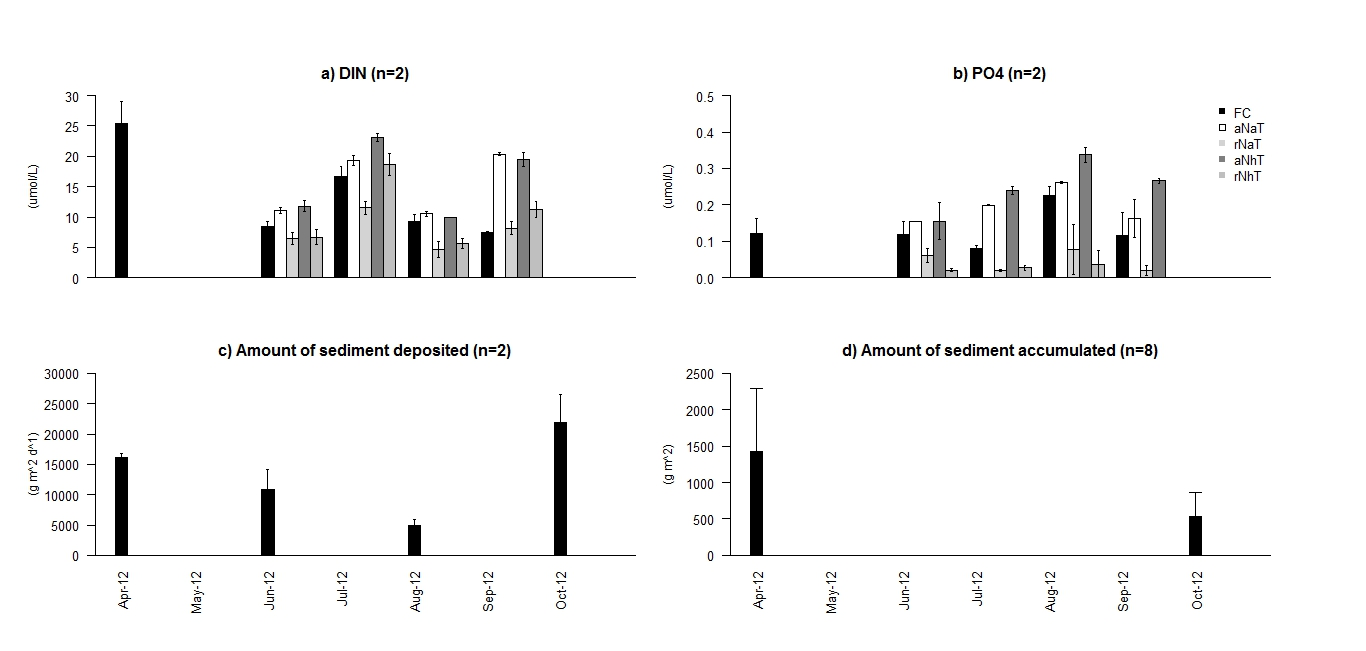

Supplement: S1 Fig — The sediment [34] (S1 Table) and nutrient [28] (S2 Fig.) data collected at the site were compared with other data from Monte Conero region collected between 2008 and 2011. Gaps are months in which the data was not collected. Codes are: FC = field control; aNaT = ambient nutrients, ambient temperature; rNaT = reduced nutrients, ambient temperature; aNhT = ambient nutrients, high temperature; rNhT = reduced nutrients, high temperature. (TIF) [file pone.0120837.s001.tif]

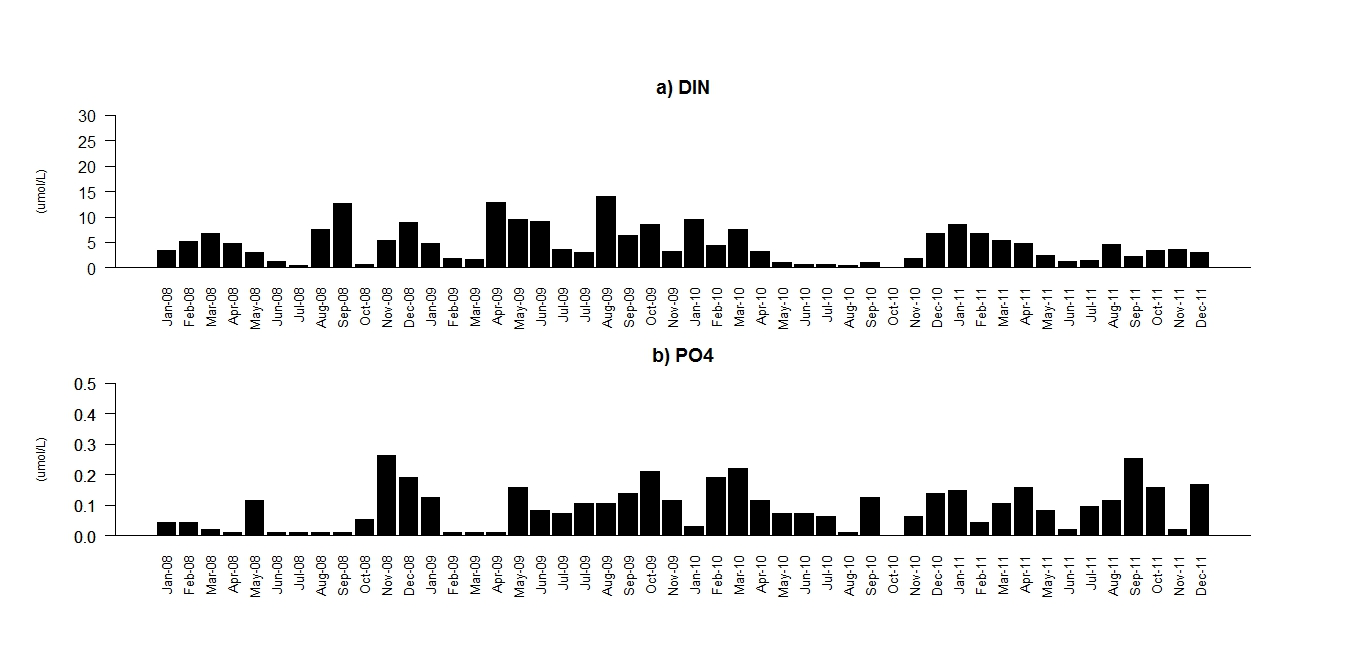

Supplement: S2 Fig — Gaps are missing data. Data was obtained from ARPA Marche, courtesy of G. De Grandis. (TIF) [file pone.0120837.s002.tif]

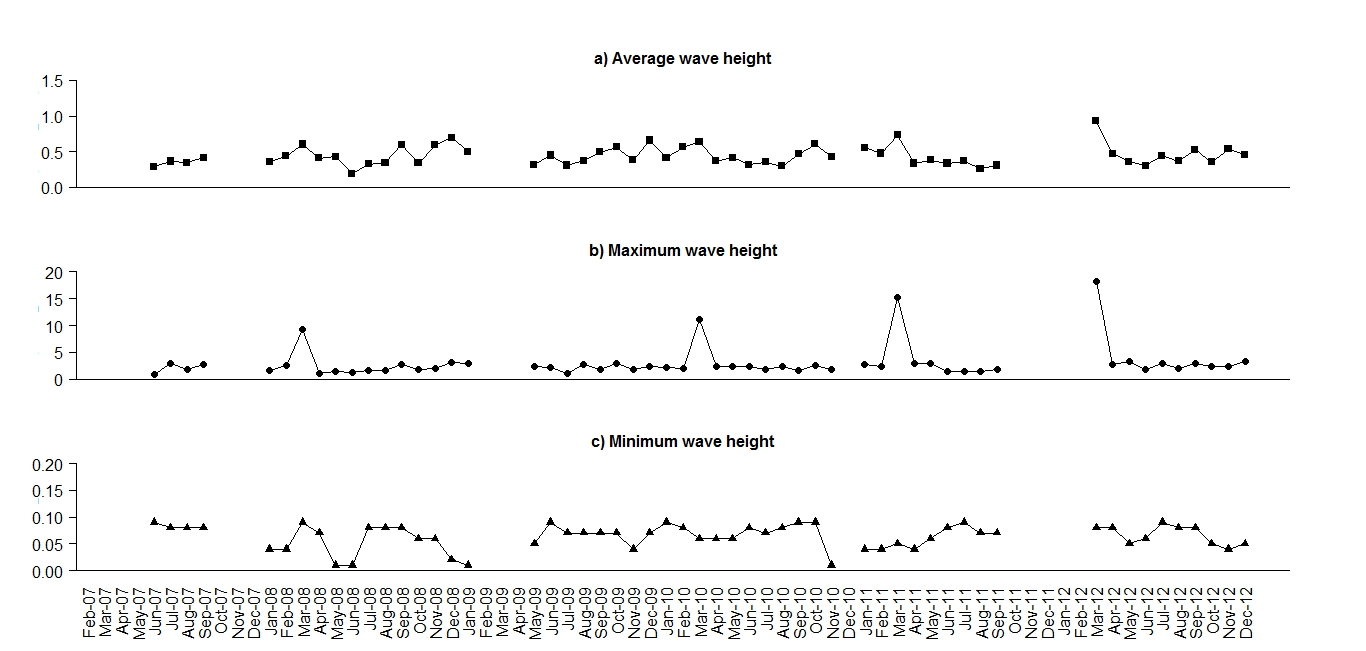

Supplement: S3 Fig — Data was downloaded from ARPA Emilia Romagna via http://dexter-smr.arpa.emr.it/. Gaps are missing data. (TIF) [file pone.0120837.s003.tif]

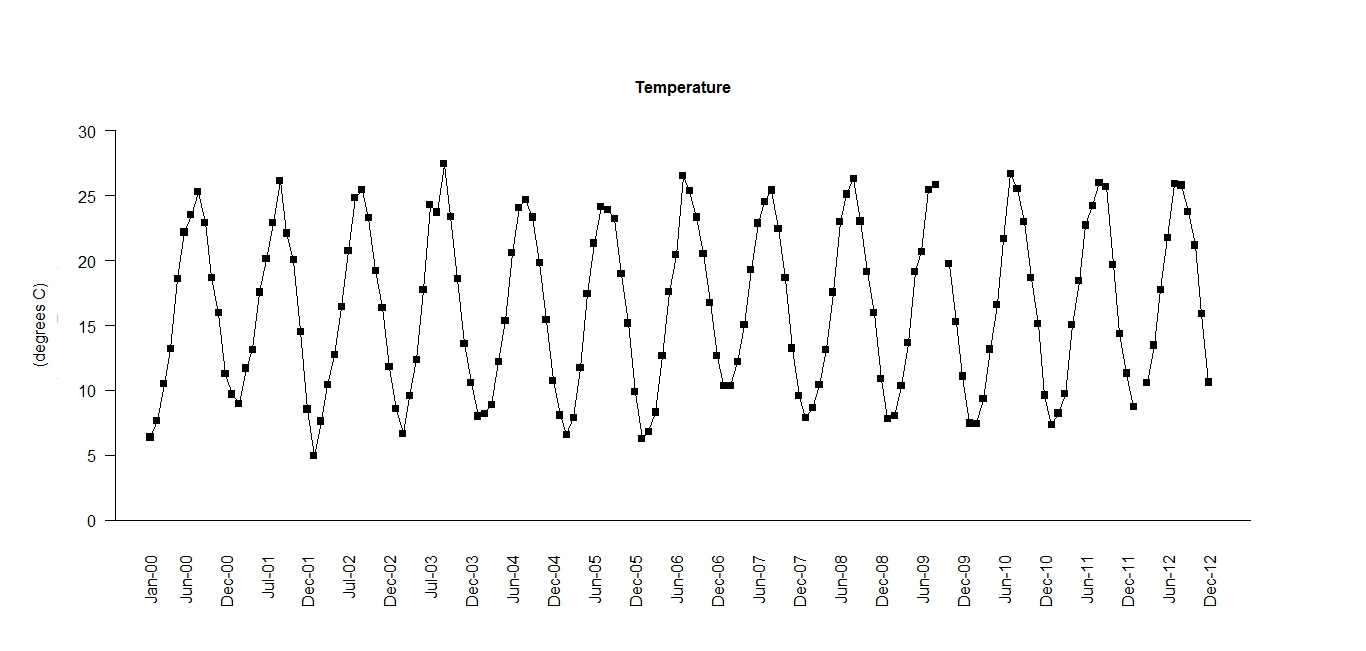

Supplement: S4 Fig — Data was obtained from ISPRA, courtesy of Dr G. Sara. Gaps are missing data. (TIF) [file pone.0120837.s004.tif]
