# Supplementary material for: Management of Local Stressors Can Improve the Resilience of Marine Canopy Algae to Global Stressors
Source: PLoS One. 2015 Mar 25;10(3):e0120837. doi: 10.1371/journal.pone.0120837 (PMC4373769; doi:10.1371/journal.pone.0120837)
Supplement: S1 Table — Measurements were taken in summer of 2009. Data obtained courtesy of Dr S. Perkol-Finkel. (DOCX) [file pone.0120837.s005.docx]

|  | Monte Conero Site 1 | Monte Conero Site 2 | Rovinj Site 1 | Rovinj Site 2 | Corsica Site 1 | Corsica Site 1 |
| --- | --- | --- | --- | --- | --- | --- |
| Accumulated  (g/m^2^)  N=10 | Mean=  1683.665  SE=  Max=  3400 |  |  |  |  |  |
| Deposited  (g/m^2^/day)  N=4 | Mean=  269.8577  SE=74.375  Max=334.542 | Mean=  129.8477  SE=22.944  Max=155.345 | Mean=  30.028  SE=7.817  Max=38.977 | Mean=  41.269  SE=13.228  Max=54.58 | Mean=  81.857  SE=  122.5872  Max=  265.5225 | Mean=  34.915  SE=  14.37384  Max=44.892 |
